# Triple super phosphate impact on maize root hydraulic conductance

**DOI:** 10.1093/aob/mcaf266

**Published:** 2025-10-22

**Authors:** Nahid Jafarikouhini, Thomas Sinclair, Amanda Cardoso, Luke Gatiboni, Thomas Rufty

**Affiliations:** Crop and Soil Sciences Department, North Carolina State University, Raleigh, NC 27695, USA; Crop and Soil Sciences Department, North Carolina State University, Raleigh, NC 27695, USA; Crop and Soil Sciences Department, North Carolina State University, Raleigh, NC 27695, USA; Crop and Soil Sciences Department, North Carolina State University, Raleigh, NC 27695, USA; Crop and Soil Sciences Department, North Carolina State University, Raleigh, NC 27695, USA

**Keywords:** Hydroponic solution, maize, pH, root hydraulic conductance, transpiration, triple super phosphate

## Abstract

**Background and Aims:**

The discovery that application of triple super phosphate (TSP) to near-neutral pH soils induced plant drought resilience opened the possibility of a new approach to managing crops for water-deficit conditions. A key component of the TSP response appeared to be decreased root hydraulic conductance. In the current study, maize plants were grown on hydroponic solutions to investigate characteristics of root hydraulic conductance response to TSP.

**Methods:**

Maize (*Zea mays* L.) was grown on hydroponic solutions to which responses to pH, TSP and H_2_PO_4_**^−^** concentrations, and timing of changes in solution pH were determined. Root hydraulic conductance was determined for seedlings by placing them in a pressure chamber and measuring sap exudation for 1 h with the root system subjected to 0.2 MPa atmospheric pressure.

**Key Results:**

Root hydraulic conductance decreased to approximately 40 % of the value for TSP-grown plants at solution pH 6.75 as compared to plants grown with diammonium phosphate. Also, hydraulic conductance decreased when solution concentrations of TSP and of H_2_PO_4_**^−^** were increased. A test of the temporal response to solution pH change showed root hydraulic conductance altered from the unchanged pH value required at least 2–4 d, indicating longer-term response to pH treatment than purely a biochemical response.

**Conclusions:**

These results quantified specific conditions under which TSP caused decreases in root hydraulic conductance in the studied maize hybrid. Decreased root hydraulic conductance observed in response to TSP and near-neutral pH was congruent with previous observations of TSP-induced decreases in soil water extraction at elevated soil water content, which has been identified as a basis for crop drought resilience.

## INTRODUCTION

Soil water deficit is a common stress experienced by maize grown in the USA. A major increase in probability of yield increase has been identified in crop genotypes that expressed decreased transpiration rate at high soil water content during soil drying (Sinclair *et al.*, 2010). One alternative to achieve expression of initiation of transpiration decrease at higher soil water content would be the introduction of a crop management scheme that achieves such a response. One such possibility for alternative management for maize could be the application of triple super phosphate [TSP, Ca(H_2_PO_4_)_2_] to the soil. Previously, the unexpected discovery was reported that application of TSP to soils resulted in initiation of a decrease in transpiration rate of maize (*Zea mays* L., hybrid DKC 67-70) plants at a higher soil water content than with application of diammonium phosphate [DAP, (NH_4_)_2_(HPO_4_)] (Sinclair *et al*., 2024; 2025*b*). Such a response to TSP caused the plants to have lower transpiration rates earlier than the DAP treatment as the soil dried so that there was a temporal conservation of soil water with the TSP treatment. The soil water conservation resulted in delayed development of soil water deficit, which resulted in TSP-treated maize plants being more drought resilient than those supplied with DAP application (Sinclair *et al*., 2024). Significantly, the drought-resilience characteristic resulting from initiation of a decrease in plant water extraction at elevated soil water content has been found in a peanut (*Arachis hypogaea* L.) genotype resulting in greater productivity under dryland conditions (Sinclair *et al*., 2018).

The mechanistic basis and conditions for induction of drought resilience by TSP in the previously studied maize hybrid is unknown. While it has been found in sorghum (*Sorghum vulgare* Pers.) that the absence of phosphorus in a nutrient solution resulted in a substantial decrease in root hydraulic conductivity (Shangguan *et al*., 2005), a quantitative response specifically to TSP has not been reported. However, there are a few studies of the temporal response of transpiration rate to pH changes of Hoagland’s solution, which provided H_2_PO_4_^−^ as the source of phosphorus. In a study with roots of maize (Ehlert *et al*., 2009) and of three tree species (Zhang *et al*., 2013) the half-life in the decrease in transpiration rate with increase in treatment pH was approximately 15 min.

A possible cause of the previously observed transpiration rate sensitivity to pH when providing H_2_PO_4_^−^ to plants may be related to root hydraulic conductance. Zhang and Zwiazek (2016) showed in paper birch (*Betula papyritera*) that increasing the pH of Hoagland’s solution from 5 to 7 to 9 resulted in major decreases in root conductance at each step. In a study by Sinclair *et al*. (2025*a*) with maize grown on pH 6.6 soil, it was found that root hydraulic conductance was much less with the application of TSP as compared to DAP. Adding a weak acetic acid solution to TSP-treated soil to decrease soil pH from 6.6 to 5.4 resulted in an increased root hydraulic conductance and a lowering of the fraction of transpirable soil water (FTSW) threshold for initiation of transpiration decrease from 0.582 to 0.435 (Sinclair *et al*., 2025*a*). That is, with TSP application and lower soil pH soil there was both an increase in root conductance associated with water uptake and a substantial loss of the plant water conservation characteristic induced by TSP.

There appears to be no direct evidence supporting the hypothesis that root hydraulic conductance with TSP application is sensitive to the solution pH to which roots are exposed. The objective of the current study was to examine the TSP sensitivity of maize root hydraulic conductance to various treatments of nutrient solutions to which roots were exposed. A series of four experiments with nutrient solutions is reported in which root hydraulic conductance was determined. The first experiment was simply to document the root hydraulic response to pH for solutions containing either TSP or DAP. The second and third experiments were to document the response of root hydraulic conductance to TSP concentration and to dihydrogen phosphate (H_2_PO_4_**^−^**) concentration supplied as ammonium dihydrogen phosphate. The fourth experiment determined the temporal response in root hydraulic conductance to changes in solution pH. The hypothesis for this fourth experiment was that a rapid change in root hydraulic conductance after changing solution pH would probably indicate a rapid biochemical response to solution pH.

## MATERIALS AND METHODS

### Root hydraulic conductance determination

To understand the previous report of plant drought resilience induced by TSP, studies using maize hybrid DKC 67-70 (the same hybrid as used in the study of Sinclair *et al*., 2024) were undertaken. Measurements of root hydraulic conductance were made by growing plants in small-volume PVC pots (∼400 mL nutrient solution; 5 cm in diameter, 27 cm tall) that could be placed in a pressure chamber. Seeds were imbibed in nutrient solution treatments appropriate for each experiment. After germination, seeds were individually placed in foam and inserted in the top of each pot containing the treatment hydroponic solution. During plant growth, air was continuously bubbled through the solution in each pot at about 1 L min^−1^ to avoid plant symptoms of oxygen deprivation.

The fundamental hydroponic solution for this study (Table 1) was the solution used by the North Carolina State University Phytotron (Saravitz *et al*., 2009). Modifications were made in the original solution to have the elemental concentrations equal for the TSP and the DAP treatments: phosphorus (1 mmol L^−1^), ammonium (2 mmol L^−1^), nitrate (7 mmol L^−1^), potassium (5 mmol L^−1^) and calcium (2 mmol L^−1^). To achieve the desired solution pH, low-concentration solutions of calcium carbonate or acetic acid were used.

**Table 1.** Concentrations of chemicals in hydroponic solution used in triple super phosphate (TSP) and diammonium phosphate (DAP) experiments.

Chemical	Formula	TSP solution (mmol L^−1^)	DAP solution (mmol L^−1^)
Triple super phosphate	Ca(H_2_PO_4_)_2_	0.5	—
Diammonium phosphate	(NH_4_)_2_HPO_4_	—	1.0
Calcium nitrate	Ca(NO3)_2_.4H_2_O	1.5	2.0
Ammonium nitrate	NH_4_NO_3_	2.0	—
Potassium nitrate	KNO_3_	1.0	2.0
Magnesium nitrate	Mg(NO_3_).6H_2_O	1.0	1.0
Potassium sulphate	K_2_SO_4_	2.0	1.5
Iron sequestrene	10 % Fe	0.5	0.5

To document possible changes of nutrient solution pH when plants were grown on nutrient solution, a preliminary experiment was done to track changes of solution pH over time. This experiment showed that the pH of the nutrient solution decreased after more than 2 d. No precipitation in the solution was observed in this short time interval. Therefore, the nutrient solution in these experiments was changed every other day to maintain stable nutrient treatments.

Plants on the nutrient solution pots were placed in a growth room with day/night temperatures of 32/26 °C, respectively. These warm conditions were selected to mimic the warm conditions to which field-grown maize in the USA could be exposed during summer. The photoperiod was 12 h and the light level at plant height during the day was 530 µmol m^−2^ s^−1^. No differences in root size were observed visually among treatments as was previously reported for plants of this hybrid when grown on various soil treatments (Sinclair *et al*., 2024).

Once plants had produced two fully extended leaves, root hydraulic conductance was determined following the protocol given by Sinclair and Jafarikouhini (2022). A hydroponic pot with intact roots still immersed in the treatment nutrient solution was placed in a pressure chamber with the shoot protruding from the chamber. The shoot was cut from the plant approximately 2 cm above the stopper that was used to seal the plant in the pressure chamber. A silicone tube filled with cotton was placed over the stump of the stem to collect exuded sap. Air was used to pressurize the atmosphere in the chamber at 0.2 MPa. Exuded sap was collected for 1 h while the plant root system was under pressure. Root conductance was calculated based on the amount of collected exuded sap resulting in units of g sap h^−1^ MPa^−1^. Since the overall procedure took approximately 90 min per plant, no more than a total of six plants were measured each day.

### Response to pH

Root hydraulic conductance was measured on plants continuously cultivated on hydroponic solution treatments of either TSP or DAP and pH adjusted to 5.75, 6.0, 6.25, 6.5 or 6.75. Since the solution was initially at about pH 6.0, calcium carbonate or acetic acid were used to adjust solution pH. Root hydraulic conductance was measured for three replicate plants for each combination of phosphorus formulation and pH.

### Response to TSP concentration

The previous pH experiment showed the lowest root hydraulic conductance for the TSP treatment among the various pH treatments was 6.75. Therefore, the aim of this study was to document the root hydraulic conductance values in response to TSP concentration and solution pH of 6.75. The tested TSP concentrations were 0.1, 0.2, 0.3, 0.5, 0.7 and 1.0 mmol L^−1^. Six replicate plants were measured for each TSP treatment.

### Response to H_2_PO_4_^−^ concentration

In this experiment, possible root hydraulic conductance sensitivity to the H_2_PO_4_**^−^** component of TSP was measured by replacing TSP in the nutrient solution with ammonium dihydrogen phosphate, NH_4_(H_2_PO_4_). The concentration of NH_4_(H_2_PO_4_) was varied among 0.2, 0.4, 0.6, 1.0, 1.4 and 2.0 mmol L^−1^ to align with the H_2_PO_4_**^−^** concentrations in the previous TSP experiment. The pH of the solutions was set at 6.75.

Since calcium was not being provided by TSP in this experiment, the concentration of Ca(NO_3_)_2_.4H_2_O in the hydroponic solution was increased from 1.5 mmol L^−1^ of the standard solution to 2 mmol L^−1^. A further adjustment in the hydroponic solution was made to the ammonia nitrate concentration to account for the differences in ammonium added by the different NH_4_(H_2_PO_4_) treatments. That is, with increasing NH_4_(H_2_PO_4_) treatment concentration the concentration of ammonia nitrate was decreased to achieve the same solution ammonium concentration. All other nutrients were the same as given in Table 1. Root hydraulic conductance was determined for six replicate plants for each NH_4_(H_2_PO_4_) treatment.

### Response to temporal change in pH

The previous experiments demonstrated that root hydraulic conductance was sensitive to both phosphorus formulation and solution pH. It was, however, unknown how quickly root hydraulic conductance changed in response to a change in solution pH. In this temporal experiment, the overall duration of plant growth on standard hydroponic solutions of TSP or DAP was the same as done in previous experiments with the initial solution pH at either 5.75 or 6.75. On days 2 or 4 before root hydraulic conductance was determined, the hydroponic solution was changed to expose the roots to pH 6.75 in the case of an original pH 5.75 treatment and to pH 5.75 in the case of an original pH 6.75 treatment. The change in solution pH was done to gain possible insight into the rapidity in root hydraulic conductance adjustment in response to solution pH. Three replicate plants were included in each of the eight treatment combinations (2 solution pH changes × 2 initial pH levels × 2 phosphorus formulations).

## RESULTS AND DISCUSSION

### Response to pH

**Fig. 1.**
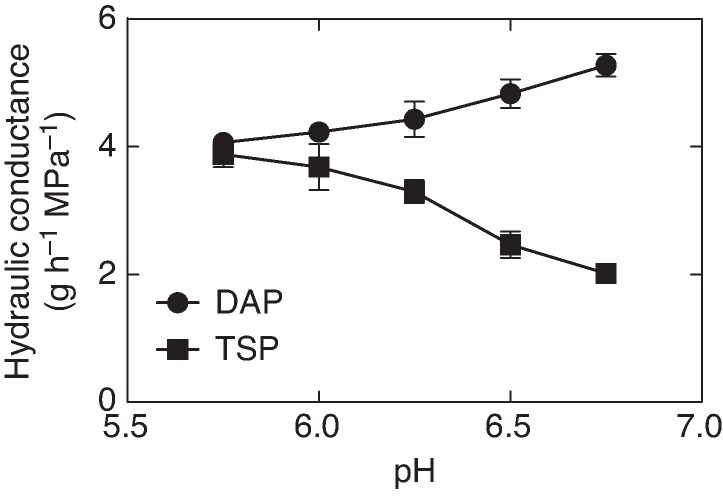
Root hydraulic conductance versus solution pH for triple super phosphate (TSP) and diammonium phosphate (DAP) treatments of 0.5 mmol L^−1^. Error bars represent the standard deviation of each datum (*n* = 3).

Root hydraulic conductance for both TSP and DAP was sensitive to the pH of the hydroponic nutrient solution (Fig. 1). At pH 5.75, the root hydraulic conductance of plants grown on either TSP or DAP solutions was equal. The root hydraulic conductance of the two phosphorus formulations diverged substantially, however, as pH increased. Root hydraulic conductance decreased with increasing pH for the TSP treatments while for the DAP treatments root hydraulic conductance increased with increasing pH. At pH 6.75, root hydraulic conductance of the TSP treatment was only about 40 % that of the DAP treatment.

The response in root hydraulic conductance with hydroponic solutions was consistent with previous experiments done with this maize hybrid on soils of differing pH. Previously, a comparison was made of root hydraulic conductance between native soils with a pH of about 6.5 and native soil with pH adjusted to 6.0–6.1 with a weak acetic acid solution (Sinclair *et al*., 2025*b*). TSP applied to these soils resulted in a lower root hydraulic conductance when the soil was at the high pH as compared to the low pH treatment. In those previous experiments with soil adjusted to a low pH of 5.4, root hydraulic conductance was nearly equivalent between the TSP and DAP treatments.

The decrease in root hydraulic conductance with TSP and increasing pH (Fig. 1) would be consistent with previous reports of limiting root water uptake at a higher soil water content (Sinclair *et al*., 2025*a*, *b*). The combination of low root hydraulic conductance with TSP and decreasing soil hydraulic conductance as the soil dries could cause the observed initiation of decrease in transpiration rate at a higher soil water content. That is, the application of TSP on higher pH soils potentially has the capacity to cause the initiation of soil water conservation at a higher soil water content, resulting in increased crop drought resilience.

A somewhat decreased root hydraulic conductance with DAP treatment was also observed for this maize hybrid when plants were grown on soils with lowered pH (Fig. 1), which would be expected to result in soil water conservation. This predicted result was consistent with the observed increase in the soil water content threshold for the decrease in transpiration rate with soil drying when DAP was applied to soil of low pH (Sinclair *et al*., 2025*a*, *b*). However, under practical field management, a low soil pH can have a negative impact on plant growth, consequently lowering crop yield. Therefore, intentionally decreasing soil pH to achieve crop drought resilience with DAP would probably be inappropriate.

One hypothesis to explain root hydraulic conductance sensitivity to pH with TSP treatment could be the reported pH-dependent changes in membrane water permeability in response to pH attributed to aquaporins (Németh-Cahalan *et al*., 2004; Kapilan *et al*., 2018). However, the changes in root hydraulic conductance with TSP and variant pH observed for maize plants grown on hydroponic solution (Fig. 1) were opposite of those in membrane water permeability resulting from changes in soil pH. To achieve the opposite responses to pH reported for aquaporins and that for TSP-hydroponic solutions would require remarkable reversals in solution pH in the solution flow in the root from the cortex into the xylem. An alternative to the aquaporin pH-dependent hypothesis is required to explain decreasing root hydraulic conductance with increasing pH under TSP treatment.

### Response to TSP concentration

**Fig. 2.**
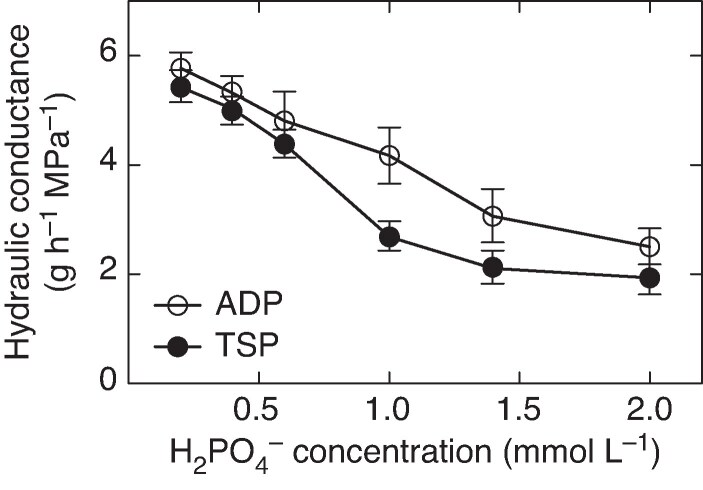
Root hydraulic conductance versus various concentrations of triple super phosphate (TSP) and ammonium dihydrogen phosphate [ADP, NH_4_(H_2_PO_4_)] shown graphically as mmol H_2_PO_4_**^−^** L^−1^. Error bars represent the standard deviation of each datum (*n* = 6).

The experiment with pH 6.75 hydroponic solutions and differing TSP concentrations showed substantial sensitivity in root hydraulic conductance to solution TSP concentration (Fig. 2). There was a progressive decline in root hydraulic conductance from the lowest tested TSP concentration (0.1 mmol L^−1^, equivalent to 0.2 mmol H_2_PO_4_^−^ L^−1^) to the highest TSP concentration (1.0 mmol L^−1^). The decline was not linear with TSP concentration, but rather a sigmoid-type response. The decrease in root hydraulic conductance was highest in the transition from TSP concentration of 0.3–0.5 mmol L^−1^ (equivalent to 0.6–1.0 mmol H_2_PO_4_**^−^** L^−1^).

These results clearly showed that the solution TSP concentration had to be at least at a minimum level to result in a decrease in root hydraulic conductance that could result in an altered pattern of soil water extraction with soil drying. Based on this hydroponic experiment, a TSP solution concentration of at least 0.5 mmol L^−1^ (1.0 mmol H_2_PO_4_**^−^** L^−1^) would be required in the soil to maximize water conservation as a result of an initiation of a decrease in transpiration rate at a high soil water content.

### Response to H_2_PO_4_^−^ concentration

While the previous experiment with TSP in hydroponic solution clearly showed substantial sensitivity of root hydraulic conductance to solution TSP concentration, it did not resolve which component of TSP, either Ca^2++^ or H_2_PO_4_**^−^**, is the basis for this sensitivity. To explore the component of TSP that may contribute a major role in limiting root hydraulic conductance with a pH 6.5 solution, an experiment was done in which TSP was replaced with NH_4_(H_2_PO_4_). In this experiment, a concentration range of NH_4_(H_2_PO_4_) was tested while maintaining overall Ca^2+^ and NH_4_**^+^** in the hydroponic solution at constant concentrations. That is, the experiment targeted an examination of the response of root hydraulic conductance specifically to variation in H_2_PO_4_**^−^** concentration.

The results of the experiment over a range of H_2_PO_4_**^−^** concentrations were consistent with the response observed for TSP concentration (Fig. 2). At lower concentrations of H_2_PO_4_**^−^** there was relatively high root hydraulic conductance, which decreased approximately linearly as H_2_PO_4_**^−^** concentration was increased. These results indicate that the H_2_PO_4_**^−^** component of TSP is the main active component associated with the transpiration response on drying soil induced by TSP, which is consistent with the previous reports (Ehlert *et al*., 2009; Zhang *et al*., 2013) of root hydraulic conductance variation with plants grown on Hoagland’s nutrient solution.

### Response to temporal change in pH

Given the high sensitivity of the TSP response to solution pH (Fig. 2), an experiment was undertaken to establish the temporal sensitivity of root hydraulic conductance to a change in solution pH. The hypothesis for the experiment was that a fairly rapid response in root hydraulic conductance to pH change was likely to indicate a short-term, biochemically pH-sensitive process. Ktitorova and Skobeleva (2008) reported for barley seedlings (*Hordeum vulgare* L.) grown with potassium phosphate that changing solution pH from 6.0 to 5.0 with acetic acid caused a 48 % decrease in root hydraulic conductance in 15 min as measured by root diameter change in response to solution osmotic potential.

**Fig. 3.**
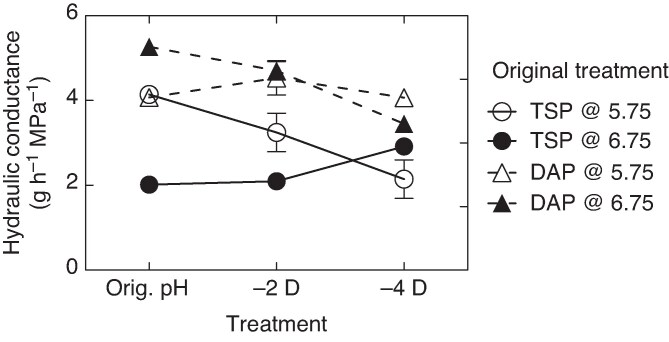
Root hydraulic conductance for triple super phosphate (TSP) and diammonium phosphate (DAP) treatments subjected to temporal pH treatments (original, continuous pH treatment, and switch in pH treatments between 5.75 and 6.75 at 2 and 4 d prior to measurement of root hydraulic conductance). Error bars represent the standard deviation of each datum (*n* = 3) when greater than the size of the symbol.

To gauge the root hydraulic conductance response time of this maize hybrid under the conditions in which we measured root hydraulic conductance, three temporal treatments of pH were applied in the nutrient solution. As shown previously (Fig. 1), root hydraulic conductance values for the TSP and DAP solutions at pH 5.75 in the current experiment were equal (Fig. 3). Switching the DAP treatment to pH 6.75 for up to 4 d before root hydraulic conductance measurement had no influence on root hydraulic conductance. Switching the TSP treatment from pH 5.75 to pH 6.75 caused a decrease in root hydraulic conductance but the full adjustment in conductance took 4 d. The case where solution pH was switched from 6.75 to 5.75 also showed a slow adjustment in root hydraulic conductance for both TSP and DAP. Two days on the lower pH solution had little influence on root hydraulic conductance and only after 4 d were there major adjustments in root hydraulic conductance.

These results examining the temporal response of root hydraulic conductance to change in pH (Fig. 3) indicated a slow adjustment in hydraulic conductance of intact roots following a change in solution pH (Fig. 3). The adjustment does not seem to support a rapid biochemical adjustment in the mechanism associated with root water uptake. Rather, these results appear to indicate a long-term biochemical sequence or a structural adjustment in the roots that takes several days after imposing an altered solution pH.

## CONCLUSIONS

The current study was undertaken to gain insight into previous observations of the sensitivity of root hydraulic conductance to soil pH with the application of TSP to the soil (Sinclair *et al*., 2025*a*, *b*). For consistency with these previous reports, the same maize hybrid as used in those studies was used in the hydroponic studies reported in this paper. Consequently, some caution is required in extending these results to other maize hybrids. Also, this study was done with young plants so extrapolation of the sensitivity of more developed maize plants to TSP and soil pH was unresolved. Nevertheless, increasing the pH of TSP hydroponic solution up to pH 6.75 was clearly associated with decreasing root hydraulic conductance (Fig. 1). Further, increasing the concentration of TSP and of H_2_PO_4_**^−^** in nutrient solutions of pH 6.75 resulted in large decreases in root hydraulic conductance (Fig. 2). These observed decreases in root hydraulic conductance at pH 6.75 are consistent with the hypothesis that the initiation of a decrease in transpiration rate at high soil water content with higher pH soils is linked to decreased root hydraulic conductance.

The underlying process for the sensitivity of root hydraulic conductance to pH and solution H_2_PO_4_^−^ was not resolved in the current study. However, the experiments showed that root hydraulic conductance response to changes in solution pH occurred over several days, indicating the adjustment does not seem to be linked directly to a rapid biochemical change induced by solution pH (Fig. 3).

Overall, the results of this study were congruent with the conclusion that solution phosphorus formulation and pH can have a major influence on root hydraulic conductance in the studied maize hybrid. In particular, the solution to which roots are exposed that contains TSP and has a pH at 6.5 or greater induced low root hydraulic conductance. These observations of root hydraulic conductance are consistent with soil observations with TSP on high pH soil of an initiation of a decrease in transpiration rate at high soil water content on drying soil resulting in the possibility of crop drought resilience. Future studies are clearly required with other maize hybrids and in field tests to fully document maize response to TSP application on high pH soil.

## Data Availability

Data will be provided upon request to the corresponding author.

## References

[mcaf266-B1] Ehlert C, Maurel C, Tardieu F, Simonneau T. 2009. Aquaporin-mediated reduction in maize root hydraulic conductivity impacts cell turgor and leaf elongation even without changing transpiration. Plant Physiology 150: 1093–1104. doi:10.1104/pp.108.131458 19369594 PMC2689965

[mcaf266-B2] Kapilan R, Vaziri M, Zwiazek JJ. 2018. Regulation of aquaporins in plants under stress. Biological Research 51: 4. doi:10.1186/s40659-018-0152-0 29338771 PMC5769316

[mcaf266-B3] Ktitorova IN, Skobeleva OV. 2008. Decrease in membrane hydraulic conductance of rhizodermal cells under nitrate deficit is related to acidification at the root surface. Russian Journal of Plant Physiology 55: 621–628. doi:10.1134/S1021443708050051

[mcaf266-B4] Németh-Cahalan KL, Kalman K, Hall JE. 2004. Molecular basis of pH and Ca^2+^ regulation of aquaporin water permeability. The Journal of General Physiology 123: 573–580. doi:10.1085/jgp.200308990 15078916 PMC2234493

[mcaf266-B5] Saravitz CH, Downs RJ, Thomas JF. 2009. Phytotron procedural manual: for controlled environment research at the Southeastern Plant Environment Laboratory (North Carolina Agricultural Research Service Technical Bulletin No.244). Raleigh, NC: North Carolina State University.

[mcaf266-B6] Shangguan Z-P, Lei T-W, Saho M-A, Xue Q-W. 2005. Effects of phosphorus nutrient on the hydraulic conductivity of sorghum (*Sorghum vulgare* Pers.) seedling roots under water deficiency. Journal of Integrative Plant Biology 47: 421–427. doi:10.1111/j.1744-7909.2005.00069.x

[mcaf266-B7] Sinclair TR, Messina CD, Beatty A, Samples M. 2010. Assessment across the United States of the benefits of altered soybean drought traits. Agronomy Journal 102: 475–482. doi:10.2134/agronj2009.0195

[mcaf266-B8] Sinclair TR, Shekoofa A, Isleib TG, Balota M, Zhang H. 2018. Identification of Virginia-type peanut genotypes for water-deficit conditions based on early decrease in transpiration rate with soil drying. Crop Science 58: 2607–2612. doi:10.2135/cropsci2018.05.0293

[mcaf266-B9] Sinclair TR, Jafarikouhini N. 2022. Plant waterflow restrictions among sweet corn lines related to limited-transpiration trait. Crop Science 62: 1242–1250. doi:10.1002/csc2.20717

[mcaf266-B10] Sinclair TR, Jafarikouhini N, Pradhan D. 2024. Unexpectedly, triple super phosphate fertilizer induces maize drought resilience. Journal of Plant Nutrition 47: 1906–1915. doi:10.1080/01904167.2024.2325948

[mcaf266-B11] Sinclair TR, Jafarikouhini N, Amick A, Gatiboni L. 2025a. Effects of soil acidity and gypsum on maize drought resilience and root hydraulic conductance in response to soil fertilization with triple super phosphate. Journal of Plant Nutrition 48: 3443–3450. doi:10.1080/01904167.2025.2514716

[mcaf266-B12] Sinclair TR, Jafarikouhini N, Turner L, et al 2025b. Triple super phosphate induces soil water conservation and decreases root hydraulic conductance in maize among soils collected from six location in the United States. Plant and Soil. doi:10.1007/s11104-025-07851-3

[mcaf266-B13] Zhang W, Calvo-Polanco M, Chen ZC, Zwiazek JJ. 2013. Growth and physiological responses of trembling aspen (*Populus tremuloides*), white spruce (*Picea glauca*) and tamarack (Larix laricina) seedlings to root zone pH. Plant and Soil 373: 775–786. doi:10.1007/s11104-013-1843-5

[mcaf266-B14] Zhang W, Zwiazek JJ. 2016. Effects of root medium pH on root water transport and apoplastic pH in red-osier dogwood (*Cornus sericea*) and paper birch (*Betula papyrifera*) seedlings. Plant Biology 18: 1001–1007. doi:10.1111/plb.12483 27425790

